# Chromosomal Mosaicism in Human Feto-Placental Development: Implications for Prenatal Diagnosis

**DOI:** 10.3390/jcm3030809

**Published:** 2014-07-24

**Authors:** Francesca Romana Grati

**Affiliations:** Research & Development, Cytogenetics, Molecular Cytogenetics and Molecular Biology, TOMA Advanced Biomedical Assays S.P.A., 25/27 Francesco Ferrer Str., Busto Arsizio 21052, Varese, Italy; E-Mail: fgrati@tomalab.com; Tel.: +39-0331-652911; Fax: +39-0331-652919

**Keywords:** chromosome mosaicism, chorionic villi, mesenchyme, cytotrophoblast, amniocentesis, uniparental disomy, confined placental mosaicism, true fetal mosaicism, non-invasive prenatal screening

## Abstract

Chromosomal mosaicism is one of the primary interpretative issues in prenatal diagnosis. In this review, the mechanisms underlying feto-placental chromosomal mosaicism are presented. Based on the substantial retrospective diagnostic experience with chorionic villi samples (CVS) of a prenatal diagnosis laboratory the following items are discussed: (i) The frequency of the different types of mosaicism (confined placental, CPM, and true fetal mosaicisms, TFM); (ii) The risk of fetal confirmation after the detection of a mosaic in CVS stratified by chromosome abnormality and placental tissue involvement; (iii) The frequency of uniparental disomy for imprinted chromosomes associated with CPM; (iv) The incidence of false-positive and false-negative results in CVS samples analyzed by only (semi-)direct preparation or long term culture; and (v) The implications of the presence of a feto-placental mosaicism for microarray analysis of CVS and non-invasive prenatal screening (NIPS).

## 1. Introduction

At the time of the first diagnosis of fetal chromosome abnormality in chorionic villi in 1983 [1], the general assumption was that the chromosome constitution of placenta reflected the true fetal karyotype. Soon after, a great deal of effort focused on investigating and understanding the genetics related to chorionic villi. In particular, fetal-placental discrepancies, that were responsible for false positive and negative results were evident, and the uniparental disomy (UPD) condition generated by embryo rescue emerged. These events were considered important mechanisms during early stages of the embryo development so that the risk of erroneous diagnosis became relevant [2,3,4,5,6]. To reduce the risk of false results, the cytogenetic diagnosis of chorionic villous samples (CVS) was established, combining direct method incubation (or semi-direct/short term culture, STC) with long-term culture (LTC). Nevertheless, in some situations a confirmatory amniocentesis emerged as necessary to elucidate the true fetal chromosome status [7,8]. 

In this review, the mechanisms underlying chromosomal mosaicism are described. Because the TOMA laboratory can take advantage of a wide cohort of first trimester cytogenetic analyses (52,673 in total), the frequency of the different types of mosaicism, the risk of fetal confirmation stratified by chromosome abnormality and placental tissue involvement, and the frequency of UPD for imprinted chromosomes associated with confined placental mosaicism (CPM) were evaluated. The incidence of false-positive and false-negative results in those cases analyzed by only one of the two cytogenetic methods mentioned above (STC or LTC) were also evaluated. Finally, the possible implications of feto-placental mosaicism for microarray analysis of CVS and non-invasive prenatal screening are discussed. 

## 2. Mechanism of Formation of Chromosome Mosaicism

Chromosomal mosaicism is one of the main interpretative issues in prenatal diagnosis. It is a biological phenomenon that indicates the presence of two or more chromosomally different cell lines in an individual arising from a single zygote [9]. This term originates from the similarity between the pictorial composition known as “mosaic”, found for the first time in Rome around the end of the 3rd century Ante Christum, and the genetic composition of an individual derived from a single fertilized egg who has two or more populations of cells with distinct genotypes. In the past, this biomedical phenomenon was underestimated because standard analysis of 10–15 cells may not detect low level (<15%–20%) mosaicism [10]. Nevertheless, standard cytogenetics has the advantage of being able to describe the distribution of the abnormal cell line and the chromosome morphology. These data have important practical implications on counseling management, as described in the present review. 

In the recent years, improvements have been made in the field of molecular cytogenetics so that currently additional methods are available to detect mosaicism. In particular, these techniques include fluorescence *in situ* hybridization analysis (FISH), quantitative fluorescent polymerase chain reaction (QF-PCR), chromosomal microarrays (array comparative genomic hybridization, aCGH; single nucleotide polymorphism array, SNP array), and, more recently, next generation sequencing (NGS) (see paragraph 7). These technologies can bypass the need for culturing and the results can be given within the space of few working days. They have been demonstrated to be useful for the detection of low frequency cell lines that require the analysis of a large number of metaphase spreads. Applications of these methods have progressively enabled mosaicism to be detected and a significant proportion of human pathogenic conditions were found to be associated with chromosomal mosaicism (see reviews by Yourov, Vorsanova and Yurov [11], and Biesecker and Spinner [12]). 

**Figure 1:**
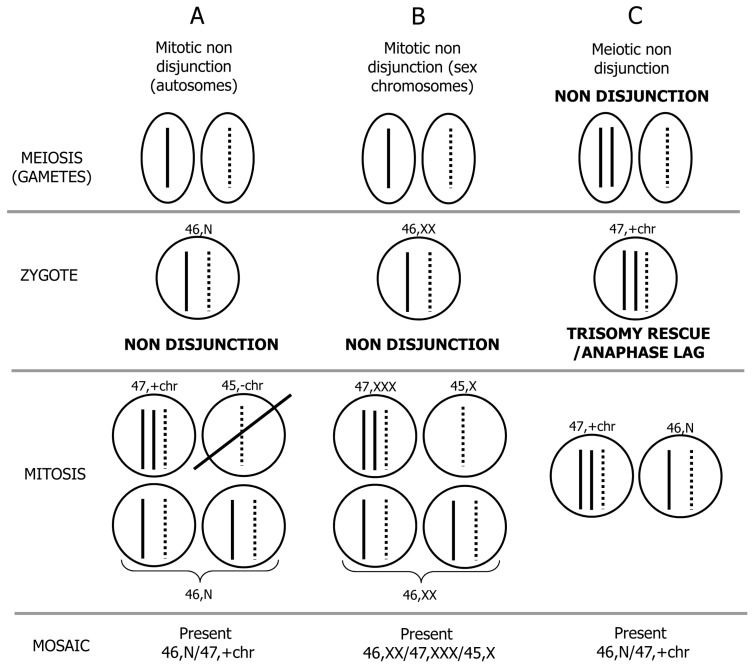
Schematic representation of mechanisms leading to chromosome mosaicism. (**A**) Mitotic non disjunction error involving an autosome: A mosaic 46,N/47,+chr is recovered in cytogenetic prenatal diagnosis; (**B**) Mitotic non-disjunction error involving a sex chromosome (X chromosome in the example): A mosaic 46,XX/47,XXX/45,X is present; (**C**) Meiotic non-disjunction error followed by trisomy rescue/anaphase lag: A mosaic 46,N/47,+chr is detectable.

Chromosomal mosaicism as diagnosed prenatally generally involves abnormal cells with full aneuploidies (usually trisomy) even if, more rarely, mosaicism for a structural rearrangement can also be found [13,14,15]. Chromosomal mosaicism in CVS and amniocytes (AF) is a well-recognized biological phenomenon occurring in 1%–2% of CVS procedures and 0.1%–0.3% of amniocentesis [8,16,17,18,19,20,21,22,23]. The underlying mechanism of mosaicism formation involves a non-disjunction (NDJ) error during a mitotic cell division or during meiosis followed by a postzygotic correction of aneuploidy. Regarding the first situation, this is the major mechanism that causes mosaicism and is gender independent [24]. This event happens in an initially normal zygote (46,N) and generates a mosaic involving 3 cell lines: the trisomic (e.g., 47,+21), the monosomic (e.g., 45,−21) and the normal cell lines (46,N) (Figure 1A). The autosomal monosomic cell line growth is usually selectively disadvantaged, and only the remaining two cell lines are retrieved during routine cytogenetic prenatal diagnosis. In case of NDJ involving an X chromosome in a 46,XX conceptus, all cell lines can expand, and the mosaic 46,XX/47,XXX/45,X is generally retrieved during routine prenatal diagnosis (Figure 1B). Regarding the second situation, when a meiotic NDJ error happens and is followed by a mitotic correction of aneuploidy, the NDJ error usually happens in maternal meiosis and give rise to an abnormal zygote (47,+chr); the normal cell line (46,N) is stored in a subsequent mitotic division with the loss of one of the extra chromosomes by either trisomy rescue or anaphase lag mechanisms (Figure 1C). The rescue mechanism was demonstrated after the introduction of CVS and DNA polymorphisms analyses when cases with trisomic villi have uniparental disomy (UPD) at confirmatory amniocentesis in the apparently normal cell line [25,26].

## 3. Postzygotic Correction of Aneuploidy and Uniparental Disomy (UPD)

**Figure 2:**
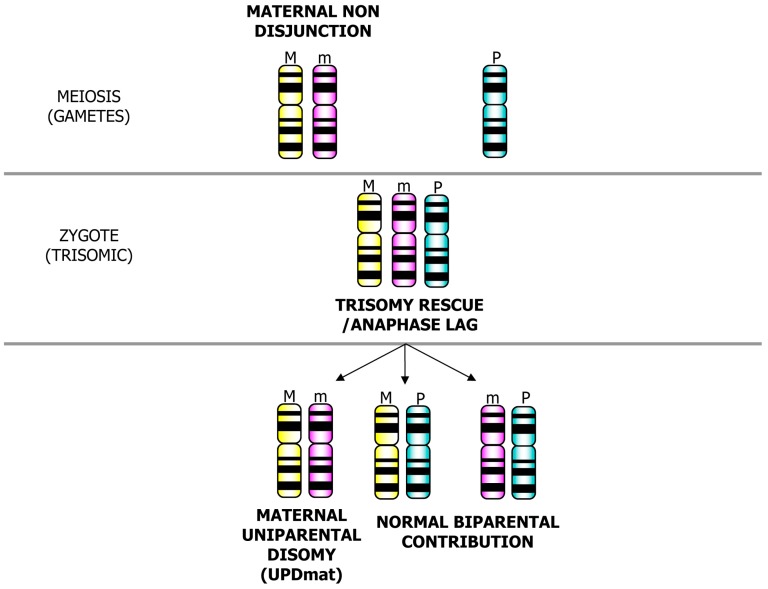
Uniparental disomy (UPD) formation after the rescue of a trisomic zygote: Trisomy rescue/anaphase lag mechanism can result in the formation of a UPD or a biparental condition.

Depending on parental origin of the extra chromosome that is lost, a biparental (one paternal and one maternal homolog) or uniparental (both homologs from one parent) disomic condition can be stored (Figure 2). 

In a diploid individual or cell line, UPD defines the presence of a chromosome pair from only one parent [27]. The uniparental origin of the homologs is of clinical interest because it can lead to the expression of recessive disorders in cases of isodisomies and when chromosomal segments are involved in UPD harbour-imprinted genes [28,29,30]. This subset of genes differs from the Mendelian expectation of inheritance because they display monoallelic (either maternal or paternal) expression based on the sex of the transmitting parent. Nearly 90 imprinted genes have, thus far, been described in humans. In humans, UPD does not cause apparent phenotypic effects when it involves most of the chromosomes. However, when UPD involves a small subset of chromosomes, it is responsible for phenotypic effects that are clinically recognizable and are usually associated with alteration of growth (intrauterine growth retardation, IUGR; postnatal growth retardation, PNGR; overgrowth; dwarfism) (see review by Miozzo and Simoni [31]). In newborns, the frequency of UPD is estimated to be 1/3500–1/5000. At least one third of UPD cases are found in association with an abnormal karyotype (a quarter of those were identified in association with mosaic or non-mosaic small supernumerary chromosome markers). There is a 1:9 rate of paternal to maternal UPD due to the higher propensity for maternal NDJ [32,33]. 

Five chromosomes have been defined as imprinted based on the associated clinical phenotypes and synteny with mouse chromosomes: chromosomes 6, 7, 11, 14, and 15. Maternally derived chromosomes 7, 11, 14, and 15 and paternally derived chromosomes 6, 11, 14, and 15 have phenotypic effects associated with a known syndrome (Table 1). 

**Table 1:** Uniparental disomies and related syndromes.

UPD type	Syndrome/Disease	OMIM reference ID	Phenotype
paternal UPD6	Transient neonatal diabete mellitus (TNDM)	#601410	IUGR, neonatal diabetes
maternal UPD7	Silver-Russell	#180860	IUGR/PNGR, dysmorfisms
maternal UPD11	Silver-Russell	#180860	IUGR/PNGR, dysmorfisms
paternal UPD11	Beckwith-Wiedemann	#130650	Overgrowth, dysmorfisms, tumors (or isolated hemihyperplasia)
maternal UPD14	Temple syndrome	*605636 and #176270	IUGR, dysmorfisms
paternal UPD14	Bell-shaped thorax, developmental retardation	#608149	Dwarfisms, dysmorfisms
maternal UPD15	Prader-Willi	#176270	Obesity, dymorfisms, MR
paternal UPD15	Angelman	#105830	MR, dysmorfisms
maternal UPD20	Growth failure, hyperactivity	*139320	IUGR/PNGR
paternal UPD20	Pseudohypoparathyroidism	*139320	Pseudohypoparathyroidism

It is highly recommended that UPD on these chromosomes be investigated in prenatal diagnosis with level II or level III mosaics (see paragraph on confined placental and true fetal mosaicisms), and analysis of AF and CVS to rule out mosaicism for trisomy or monosomy is also recommended. Chromosomes 2, 16, and 20 may also have imprinted regions, but it is unclear if their phenotypic effects are due to imprinting or to the presence of trisomic cells in the placenta and/or fetus [32,34,35,36,37]. Mitotic recombination of chromosome 20 can also give rise to UPD and type I pseudohypoparathyroidism, a situation similar to other imprinting disorders, such as Beckwith-Wiedemann syndrome or transient neonatal diabetes mellitus [38].

## 4. Detection of UPD and Discrimination of the Mechanism Generating the Uniparental Disomy Condition

**Figure 3:**
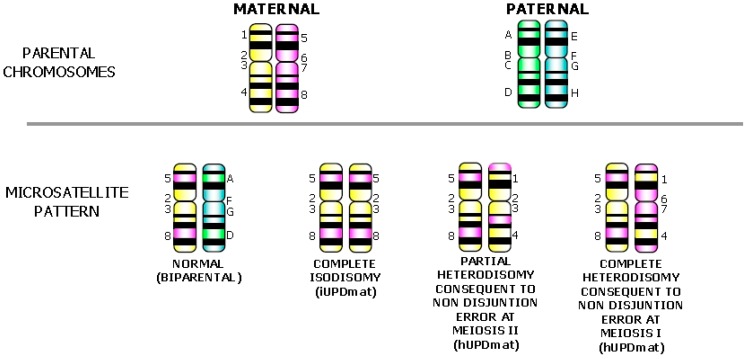
Schematic representation of the use of short tandem repeats (STRs) to determine the mechanism of formation of UPD. (**A**) Normal biparental STR profile: one paternal and one maternal allele at each informative locus is present; (**B**) Complete isodisomy due to post-zygotic reduplication of an homolog: homozygosity of all STRs markers is present; (**C**) Partial heterodisomy consequent to a non-disjunction error during meiosis II followed by trisomy rescue: homozygosity at pericentromeric STRs and heterozygosity interjected with homozygosity along chromosome arms are present; (**D**) Complete heterodisomy consequent to a non-disjunction error during meiosis I followed by trisomy rescue: informative STRs, pericentromeric and the p- and q-arms are all heterozygous and derived from only one parent.

Segregation analysis of pericentromeric short tandem repeats (STRs) markers of parents and fetus allows the discrimination of chromosomal segregation errors during meiosis (I or II) and mitosis and the detection of UPD; the STRs analysis along the chromosome arm/s identifies recombination events with the presence of isodisomy or heterodisomy [39]. In complete isodisomy (iUPD), there is homozygosity of all STRs markers as a consequence of a post-zygotic chromosome duplication [40] (Figure 3). 

Complete iUPD may be due to a “monosomy rescue”, when a monosomic gamete is fertilized by a nullisomic gamete, or by “trisomic rescue” of a trisomy of postzygotic origin (Figure 1A) [39]. With the former mechanism, no mosaicism is likely to be present because monosomy is lethal; with the latter mechanism, mosaicism for the trisomic cells may be detected by cytogenetic analysis if in large enough number [10]. Complete or partial heterodisomy (hUPD) arises through a mitotic rescue event after the formation of a trisomic zygote due to NDJ error in meiosis I or II. When STRs show homozygosity at pericentromeric loci, where no recombination events are likely to happen, and heterozygosity interspersed with homozygosity at loci along chromosome arms (Figure 3), a hUPD condition consequent to a NDJ error at meiosis II with a post-zygotic rescue can be reliably hypothesized (Figure 1C). In particular, when all informative markers (pericentromeric and along the p- and q-arms) are heterozygous, a NDJ error at meiosis I with a post-zygotic rescue of the trisomy is the cause of the UPD (Figure 3). Depending on the stage and feto-placental tissue involved in postzygotic correction of the aneuploidy, isodisomic or heterodisomic profiles could also reveal the presence of a second/third STR faint peak representing the remnant original mosaic trisomic cell line. 

**Figure 4:**
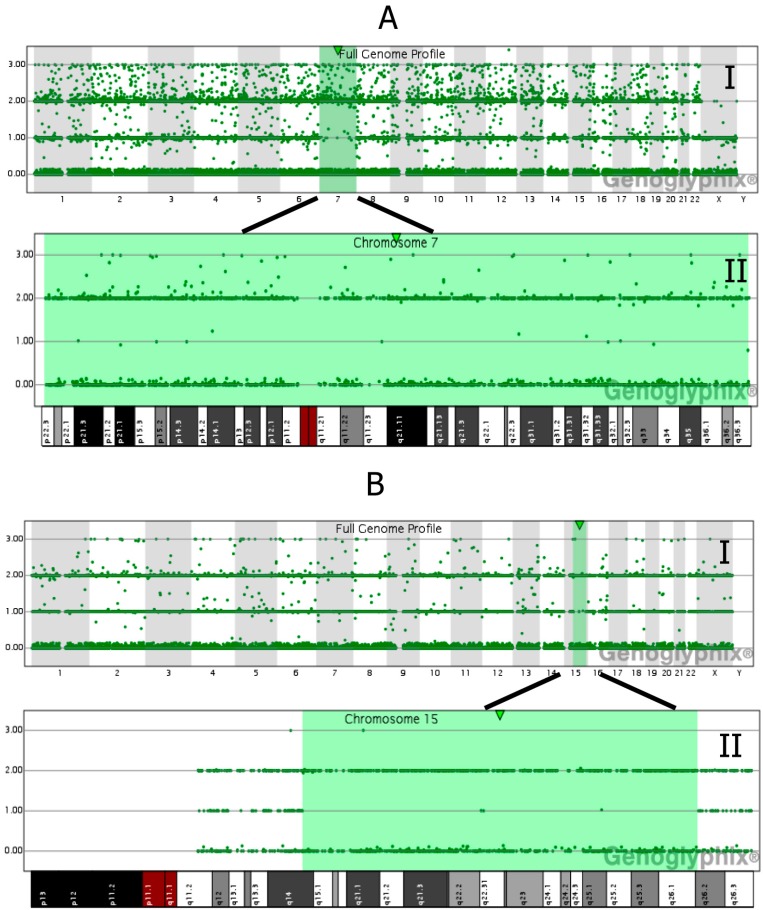
Examples of UPD detected by SNP array; (**AI**) and (**BI**): full genome profile: chromosome 7 and 15 are highlighted as regions of homozygosity (ROH) by Genoglyphix software; (**AII**) and (**BII**): complete isodisomy of chromosome 7 (**AII**) consequent to post-zygotic reduplication of an homolog and (**BII**) partial isodisomy of chromosome 15 (from q14 to q26.2) between two regions of heterodisomy consequent of a non-disjunction error during meiosis II followed by trisomy rescue are depicted (courtesy of Signature Genomic Laboratories, a subsidiary of PerkinElmer, Wallac, Turku, Finland).

SNP array should also detect the iUPD condition. In these cases, large block(s) of homozygosity (regions of homozygosity, ROH) are restricted to a single chromosome interspersed (or not) with regions of heterodisomy. Studying the parents by microarray would identify all cases of UPD, including complete hUPD [41] (Figure 4).

The first case of UPD was reported by Hubbard *et al*. [42] and involved a seven-year-old child with short stature, cystic fibrosis (CF) and growth hormone deficiency; CF was explained as homozygosity for a maternal recessive allele due to maternal isodisomy of chromosome 7 (iUPD7mat). In general, the identification of UPD allowed the localization of rare recessive diseases inherited from a single carrier parent including osteogenesis imperfecta due to iUPD7mat [43], congenital chloride diarrhea associated with iUPD7pat [44], spinal muscular atrophy related to the presence of iUPD5pat [45], and Bloom syndrome in the presence of iUPD15mat [46]. There is also the possibility of paternal XY heterodisomy in the presence of transmission of X-linked recessive conditions, such as hemophilia, from a father to a son, presumably by a mechanism of gametic complementation of a XY sperm that fertilized a X-nullisomic ovum [29,35].

## 5. Confined Placental and True Fetal Mosaicisms

### 5.1. Mosaicism in Amniotic Fluid

Three different levels of mosaicism can be detected *in vitro* during cytogenetic prenatal diagnosis on amniotic fluid [47,48,49,50]. Level I mosaicism involves the observation of a single abnormal cell: with high probability, this is a cultural artifact and considered pseudomosaicism. Level II mosaicism occurs when two or more cells with the same chromosome abnormality are seen in a culture from a single flask or in a single abnormal colony derived from an *in situ* culture. The abnormality must not involve colonies from other independent cultures. Additional studies may be performed, but these cases are almost always pseudomosaicisms. Level III mosaicism is defined as the presence of two or more cells with the same chromosome abnormality that are distributed over two or more independent cultures. These cases are likely to represent true mosaicism that is present in fetal tissues [50].

### 5.2. Mosaicism in Chorionic Villi

Cells and tissues differentiation begins at the early post-fertilization stages. The notion that the fetus may be derived from only 3/64 blastocyst cells while the remaining cells give rise to extraembryonic structures is based on studies of chimeric mouse embryos and should not be presented as firm fact for human embryos. Theoretically, a mitotic error in early stage of development is more likely to occur in extrafetal than in fetal cell lineages [51,52]. However, the distribution of normal and abnormal cell lines in the fetus and in placenta depends on the stage and mechanism of formation. Level III mosaicisms can be either confined to the placenta (CPM) or generalized to the fetus (TFM, true fetal mosaicism) and therefore identifiable at amniocentesis. The confirmation in the fetus can also be performed on fetal blood sampling by cordocentesis in improving the resolution of a mosaic cell line found in an amniotic fluid culture [53,54]. Postmortem or after-birth confirmation of a mosaic condition have also been described [55,56,57]. However, throughout the present study, the phrase “fetal confirmation” is used to refer to amniocentesis. 

When trisomy rescue occurs soon after fertilization (before the differentiation of the trophoblast and the inner cell mass), the mosaic can be generalized to both placental and fetal tissues; when it occurs at a later gestational stage (after the separation of the fetal and placental compartments), the abnormal cells may be confined to the placenta (CPM) or fetus but not necessarily to both tissues [58,59]. Due to this variable and unpredictable distribution of the abnormal cell line, when a mosaic in CVS is detected during prenatal diagnosis a confirmatory karyotype on amniocytes should be performed to discriminate between a mosaic CPM or TFM condition. Karyotype from amniocentesis analyzes the genetic constitution of a heterogeneous group of cells derived from the embryonic ectoderm and amniotic ectoderm and mesoderm, and needs to be interpreted accordingly. However, the class of amniocytes that grow preferentially in cultures seems to be the amniotic mesoderm, which might more closely reflect the true embryonic state [60]. After confirmation on amniocytes, mosaicism can be classified according to the distribution of the abnormal cell line (Table 2): in CPM type I and TFM type IV, only the cytotrophoblast involves the abnormal cell line; in CPM type II and TFM type V, only the mesenchyme is involved, and in CPM type III and TFM type VI, both placental tissues are affected by the abnormal cell line [5,50,61,62]. 

**Table 2:** Incidences of the different types of mosaicisms (CPM and TFM) found after chrionic villous and amniocytes karyotyping.

Type	Nature	Trophoblast	Mesenchyme	Amniocytes	Relative frequencies
		(direct)	(culture)		
I	CPM	Abnormal	Normal	Normal	34.76% (308/886)
II	CPM	Normal	Abnormal	Normal	42.32% (375/886)
III	CPM	Abnormal	Abnormal	Normal	10.16% (90/886)
IV	TFM	Abnormal	Normal	Abnormal	1.58% (14/886)
V	TFM	Normal	Abnormal	Abnormal	5.76% (51/886)
VI	TFM	Abnormal	Abnormal	Abnormal	5.42% (48/886)

Although Daniel *et al*. [63] have reported that 10% of mosaics in CV interpreted as CPM may reflect a cryptic fetal mosaicism that might or might not have phenotypic consequence, they may be considered without phenotypic effect, even if in a few cases, the function of the placenta may be compromised, and in turn, fetal well-being may be affected [64]. Hence, a 46,XN amniotic karyotype following a mosaic CVS still needs to be interpreted with care, even if the risks for fetal involvement are low. In general, CPM of meiotic origin are likely to be associated with a risk of pregnancy complications [59,65]. However, newborns from CPM pregnancies did not differ from a control group in terms of general health, development, behavior and intrauterine growth [66]. In the former group, there was only a slightly lower postnatal growth. Nevertheless, for a comprehensive evaluation of the prognostic effect of CPM, specific attention must be paid to the presence/absence of UPD or recessive diseases in the fetus [31,35]. 

The karyotype of the two placental cell lineages can be obtained by means of STC (cytotrophoblast) [67] or LTC (mesenchyme) of the chorionic villi [68]. Over a period of 13 years, the TOMA laboratory analyzed 52,673 CVS by STC and LTC. This cohort includes previously published cases [24]. All cases underwent similar procedures using consistent evaluation criteria and procedures that are in agreement with the Italian and European guidelines as previously reported [23,69]. 

### 5.3. Risk of Fetal Confirmation by Amniocentesis of a Mosaic Abnormality: An Examination of 52,673 Chorionic Villi Samples

When a mosaicism is found in chorionic villi, it is challenging to provide objective data that enables genetic counselors to figure out the risk of fetal involvement and the clinical relevance of the revealed mosaic condition. Importantly, the genetic counselor can refine a personalized strategy of investigation in relation to the indicated chromosome abnormality and the risk of fetal involvement as determined by retrospective cytogenetic results. On these bases, a genetic counselor might recommend invasive follow-up amniocentesis.

Our investigation showed that a CV mosaicism was found in 1136 (1.81%) of 52,673 CVS analyzed. The karyotype of 886 cases was subsequently investigated on amniocytes and classified into the six different classes of mosaicism. 

As shown in Table 2, the mosaicism was confined to the placenta in 773 cases (87.2%): 34.76% type I (308/886), 42.32% type II (375/886) and 10.16% type III (90/886); in the remaining 12.8% of cases (*n* = 113), the presence of the chromosome abnormality was also confirmed in amniocytes (TFM). 

The general chance of TFM assessed on the basis of the type of mosaicism found in CV (Table 3) was 34.8% in the case of type III, 12.0% in the case of type II and 4.4% in type I. The separation of mosaic (MA) and nonmosaic abnormalities (NMA) showed that the risk of fetal confirmation increases to 28.6% in the case of an NMA in the mesenchyme alone and decreases to 27.8% in case of an MA in both placental tissues; this latter probability is 76.9% when the mesenchyme is homogeneously involved, and 31.7% when the cytotrophoblast is homogeneously involved. When an NMA is restricted to cytotrophoblasts the risk of a TFM type IV increases to 8.9% (Table 3). 

The stratification by type of chromosome abnormality (Table 4) indicates that mosaics involving 47,+mar or sex chromosome aneuploidies have the highest risk of fetal confirmation (35.8% and 31.6%, respectively), while autosomal trisomies and 46,der karyotypes have a far lower risk of TFM (6.9% and 5.2%, respectively).

**Table 3:** Probabilities of confirmation on amniocytes of Mosaic or Non Mosaic abnormal cell line considering the different combinations of the affected placental tissues.

Trophoblast	Mesenchyme	Confirmation
(direct)	(culture)	
**A**	**N**	**TFM IV/(CPM I + TFM IV) = 14/322 = 4.4%**
MA	N	7/(236 + 7) = 2.9%
NMA	N	7/(72 + 7) = 8.9%
**N**	**A**	**TFM V/(CPM II + TFM V) = 51/426 = 12.0%**
N	MA	35/(335 + 35) = 9.5%
N	NMA	16/(40 + 16) = 28.6 %
**A**	**A**	**TFM VI/(CPM III + TFM VI) = 48/138 = 34.8%**
MA	MA	20/(52 + 20) = 27.8%
NMA	MA	13/(28 + 13) = 31.7 %
MA	NMA	10/(3 + 10) = 76.9 %
NMA	NMA	5/(7 + 5) = 41.7%

TFM = True fetal mosaicism; CPM = Confined placental mosaicism; A = Abnormal; N = Normal; MA = Mosaic Abnormality; NMA = Non Mosaic Abnormality.

Trisomy involving chromosomes 1, 4, 5, 6, 10, 11, 12, 14, 17, 19, and 22 are those with the lowest frequency (≤10 cases in the total cohort); trisomies for chromosomes 3, 9, 15, 16, and 20 showed an intermediate incidence of mosaicism (between 11 and 25 cases in the overall cohort), and the remaining chromosomes (2, 7, 8, 13, 18, and 21) showed a frequent involvement in mosaicism (>25 cases). In general, the highest fetal confirmation rate of all trisomies is present when they are generalized to the placenta with an homogeneously affected mesenchyme (66.7%). Fetal confirmation was detected for trisomies 4 and 12 with a risk of confirmation of 25% and 10%, respectively; for trisomies 8, 16 and 20 with a confirmation rate of 7.7%, of 11.8%, and of 8%, respectively; and for chromosomes 13, 18 and 21 with a risk of fetal involvement of 2.6%, 17.4%, and 31.4%, respectively (Table 4). Among all these trisomies, only T13 and 21 can be confirmed as TFM type IV. The specific risks of confirmation in amniocytes of T13, T18, and T21 are reported for the different combinations of placental tissue involvement and distribution of the abnormal cell line. Trisomy 13 has a specific risk of confirmation of 5% only when an MA is confined to cytotrophoblasts (TFM type IV). The majority of mosaic T18 cases involve only mesenchymes (CPM type II/TFM type V), showing a preferential pattern of occurrence of mosaic T18 with specific risks of fetal confirmation of 83.3% and 4% when the abnormality is present in the NMA or the MA form, respectively. When a T18 MA is confined to cytotrophoblasts the risk of TFM type IV is extremely low, while in presence of a placental generalized mosaicism, the risk for TFM type VI is 40% (2/5). Similar to T18, T21 also shows a higher prevalence of mosaics confined to the mesenchyme, with specific risks of confirmation of 60% and 15.4% when NMA and MA T21 are present in mesenchyme, respectively. When NMA T21 is confined to cytotrophoblasts there is a risk of TFM type IV of 33.3%. In presence of a placental generalized mosaicism, the risk for TFM type VI is 72.7% (8/11); this risk increases to 100% when T21 is in non-mosaic form in mesenchymes.

In the trisomy 4 TFM case, the trisomic cell line was homogeneously present in cytotrophoblasts and in mesenchymes. Due to the low survival rate of conceptuses with this homogeneous abnormality [70,71,72] over the first trimester, at 17th week of gestation an amniocentesis was performed to assess the fetal karyotype that was as follows: mos 47,XX,+4[2]/46,XX[68]. 

An opposing behavior is evident for trisomy 4 and 12 and for trisomy 2 and 7: the former are infrequently involved in mosaic conditions in CVS (≤10 cases in the overall cohort); nevertheless, they show a remarkable risk fetal confirmation. The latter are the most frequent mosaics (64 cases each one), but no TFM cases were detected.

Trisomies 2, 8, 9, 12, 17, 18, and 21 seem to have a preferential pattern of occurrence restricted to the mesenchyme; trisomies 3, 5, 11, 13 and 15 are restricted to the cytotrophoblast; trisomies 4, 7, 10, 14, 16, 20 and 22 are generalized to both placental tissues. In agreement with Wolstenholme [73], in the present study, 89% of mosaicisms for trisomy 2 and 66.7% of mosaicisms for trisomy 8 showed the abnormal cell line only in mesenchymes (57/64 and 16/24, respectively); in the majority, the trisomy was in mosaic form (48/57, 84% and 14/16, 87.5%). In line with Wolstenholme [73], in 92% of cases of mosaic trisomy 3 (23/25), the abnormal cell line was observed only in mesenchymes; the majority (20/23, 87%) showed a mosaic abnormality. A proposed explanation for the excess of trisomies 2 and 8 in mesenchymes and of trisomy 3 in cytotrophoblasts is that they are lethal if a significant number of cytotrophoblastic or mesechymal cells, respectively, are abnormal [73]. 

Mosaic multiple trisomies were present only as CPM without any fetal involvement and with a preferential pattern of occurrence restricted to the mesenchyme. 

Trisomies 4 and 16 can be found as TFM type VI with an NMA generalized to the placenta and confirmed in the fetus in a mosaic form. In contrast, trisomy 22 and tetraploidy (92,XXXX) homogenously affected both placental tissues without any involvement of the fetus. These last two cases represent rare instances of complete feto-placental discordance (false positives) and support the necessity of a confirmatory amniocentesis, especially when a homogenous abnormality is detected in pregnancies surviving the first trimester.

Regarding sex chromosome aneuploidies, all 45,X/46,XX/47,XXX mosaicisms found in CVS were confirmed in the fetus (6/6), one as TFM type V and the remaining as TFM type VI. The overall confirmation risk of 47,XXX and 47,XXY mosaics is 40% and 42.2%, respectively. When triple X is confined to cytotrophoblasts as NMA, the risk of TFM IV is 50%; otherwise, when it is generalized to the placenta, the fetal confirmation risk is 100%. In 47,XXY mosaic cases, the confirmation rate is 100% when at least one placental tissue is homogeneously affected, and null when confined to cytotrophoblasts. Monosomy X shows an overall fetal confirmation risk of 25.7% with a remarkable rate of TFM when the 45,X cell line is present as NMA in mesenchymes (75% as TFM type V and 100% as TFM type VI). No fetal involvement was found in mosaic 47,XYY in CV.

In general, the highest fetal confirmation rate of sex chromosome aneuploidies is present when the mesenchyme is homogeneously affected (80% for TFM type V and 100% for TFM type VI). 

Among the remaining structural chromosome abnormalities, mos 47,+mar showed the highest incidence of fetal confirmation (35.8%; 19/53) [74], “mos 46,t” and “mos 45,rob” were confirmed in 14.3% of cases (5/35) and “mos 46,der” in 5/97 cases (5.2%) only as TFM type V.

**Table 4:** Risk of confirmation stratified by type of mosaicism in chorionic villi and chromosome abnormality.

Chromosome abnormality	Risk of confirmation stratified by type of mosaicism in chorionic villi [Tfm/(Tfm + Cpm)]								Risk of confirmation of each chromosome abnormality
	Placental tissue involvement								
	Only cytotrophoblast (CPMI/TFMIV)		Only mesenchyme (CPMII/TFMV)		Both placental tissues (CPMIII/TFMVI)				
	NMA	MA	NMA	MA	NMA-C/MA-M	MA-C/NMA-M	MA-CM	NMA-CM	
Trisomy 1				0/1 = 0					0/1 = 0
Trisomy 2	0/1 = 0	0/4 = 0	0/9 = 0	0/48 = 0		0/1 = 0	0/1 = 0		0/64 = 0
Trisomy 3	0/3 = 0	0/20 = 0					0/2 = 0		0/25 = 0
Trisomy 4	0/1 = 0	0/1 = 0	0/1 = 0					1/1 = 100%	**1/4 = 25%**
Trisomy 5		0/2 = 0							0/2 = 0
Trisomy 6				0/1 = 0	0/1 = 0				0/2 = 0
Trisomy 7	0/4 = 0	0/32 = 0	0/2 = 0	0/22 = 0			0/3 = 0	0/1 = 0	0/64 = 0
Trisomy 8	0/3 = 0	0/6 = 0	0/2 = 0	2/14 = 14.3%			0/1 = 0		**2/26 = 7.7%**
Trisomy 9	0/1 = 0	0/2 = 0		0/11 = 0	0/1 = 0		0/2 = 0		0/17 = 0
Trisomy 10	0/2 = 0	0/2 = 0	0/1 = 0	0/5 = 0					0/10 = 0
Trisomy 11	0/1 = 0	0/2 = 0							0/3 = 0
Trisomy 12		0/1 = 0	0/1 = 0	1/8 = 12.5%					**1/10 = 10%**
Trisomy 13	0/2 = 0	1/20 = 5%		0/10 = 0	0/4 = 0		0/3 = 0		**1/39 = 2.6%**
Trisomy 14		0/4 * = 0		0/3 = 0			0/3 = 0		0/10 = 0
Trisomy 15	0/2 = 0	0/12 = 0	0/2 * = 0	0/5 = 0	0/1 = 0		0/2 = 0		0/24 = 0
Trisomy 16	0/1 = 0	0/2 = 0	0/1 = 0	0/6 = 0	0/3 = 0		1/2 = 50%	1 */2 = 50%	**2/17 = 11.8%**
Trisomy 17				0/2 = 0					0/2 = 0
Trisomy 18		0/10 = 0	5/6 = 83.3%	1/25 = 4%	0/2 = 0	1/2 = 50%	1/1 = 100%		**8/46 = 17.4%**
Trisomy 19				0/1 = 0					0/1 = 0
Trisomy 20		0/10 = 0	1/1 = 100%	1/9 = 11.1%	0/3 = 0		0/2 = 0		**2/25 = 8%**
Trisomy 21	1/3 = 33.3%	0/6 = 0	3/5 = 60%	4/26 = 15.4%	5/7 = 71.4%	3/3 = 100%	0/1 = 0		**16/51 = 31.4%**
Trisomy 22	0/3 = 0	0/1 = 0		0/2 = 0	0/1 = 0			0/2 = 0	0/9 = 0
Multiple trisomies	0/2 = 0	0/5 = 0	0/2 = 0	0/11 = 0	0/2 = 0		0/1 = 0		0/23 = 0
All autosomal trisomies	1/29 = 3.5%	1/142 = 0.7%	9/33 = 27.3%	9/210 = 4.3%	5/25 = 20%	4/6 = 66.7%	2/24 = 8.3%	2/6 = 33.3%	33/475 = 6.9%
47,XYY		0/2 = 0							0/2 = 0
45,X	4/15 = 26.7%	3/30 = 10%	3/4 = 75%	8/33 = 24.2%	0/2 = 0	1/1 = 100%	7/16 = 43.8%		**26/101 = 25.7%**
47,XXY		0/5 = 0	1/1 = 100%	3/7 = 42.9%	1/1 = 100%	2/2 = 100%	0/1 = 0		**7/17 = 42.2%**
47,XXX	1/2 = 50%	0/4 = 0		0/1 = 0		2/2 = 100%	1/1 = 100%		**4/10 = 40%**
45,X/46,XX/47,XXX				1/1 = 100%	1/1 = 100%		4/4 = 100%		**6/6 = 100%**
All sex chromosome aneuploidies	5/17 = 29.4%	3/41 = 7.3%	4/5 = 80%	12/42 = 28.6%	2/4 = 50%	5/5 = 100%	12/22 = 37.5%		43/136 = 31.6%
45,−22				0/1 = 0					0/1 = 0
47,+mar	1/1 = 100%	3/12 = 25%	1/2 = 50%	6/26 = 23.1%	2/2 = 100%	1/2 = 50%	5/8 = 62.5%		**19/50 = 35.8%**
47,+der	0/2 = 0	0/4 = 0	0/1 = 0				0/2 = 0		0/9 = 0
46,der	0/10 = 0	0/26 = 0	0/5 = 0	3/54 = 5.6%	1/1 = 100%		1/1 = 100%		**5/97 = 5.2%**
Triploid				1/1 = 100%	1/1 = 100%				**2/2 = 100%**
Tetraploidy	0/16 = 0	0/9 = 0	0/6 = 0	0/4 = 0	0/6 = 0		0/14 = 0	0/1 = 0	0/53 = 0
47,+i(13q)		0/4 = 0		0/4 = 0					0/4 = 0
47,+i(7p)				0/3 = 0					0/3 = 0
Other §				1/4 = 25%	2/2 = 100%			3/5 = 60%	**6/11 = 54.5%**
46,t or 45,rob	0/4 = 0	0/5 = 0	2/4 = 50%	3/21 = 14.3%			0/1 = 0		**5/35 = 14.3%**
All remaining abnormalities	1/33 = 3%	3/60 = 5%	3/18 = 16.7%	14/118 = 11.9%	6/12 = 50%	1/2 = 50%	6/26 = 23.1%	3/6 = 50%	37/275 = 13.5%

^§^ = See Table 8 for description; * = UPD cases, see Table 6 and the text for description; MA = Mosaic Abnormality; NMA = Non Mosaic Abnormality; NMA-C/M = non mosaic abnormality in cytotrophoblast or mesenchyme; MA/NMA-CM = mosaic or non mosaic abnormality in cytotrophoblast & mesenchyme; CPM = confined placental mosaicism; TFM = true fetal mosaicism.

Supernumerary marker chromosomes seem to have a preferential pattern of involvement restricted to the mesenchyme. However, a risk of fetal confirmation is present for each type of placental mosaicism, with the lowest one when a MA cell line is confined to mesenchyme (23.1%) and the highest one when an NMA is retrieved in the cytotrophoblast, either in a confined or a generalized form (100%). Supernumerary i(13q) and i(7q) were always detected in mosaic form restricted to mesenchymes and the risk of fetal confirmation is extremely low. 

The majority of “mos 46,der” cases are terminal deletions that are possibly caused by cultural artifacts. Regarding apparent mosaic balanced rearrangements “mos 46,t” and “mos 45,rob”, they are generally confirmed in the fetus more frequently than the unbalanced rearrangements “mos 46,der”, and most often they were detected in a mosaic condition than in the fetus. True mosaicisms for reciprocal and robertsonian translocations are rare [75] and the newborn in question did not show an abnormal phenotype. 

Table 4 provides also an extra category of mosaics called “others” that includes cases characterized by two different mosaic abnormalities in CV and AF. Overall, these conditions are identified as TFM in 54.5% of the cases (6/11), and they are described in detail in Table 5. No CPM type I and TFM type IV are present and in CPM type III and TFM type VI, the cytotrophoblast is always homogeneously affected; in CPM type II and TFM type V, the abnormal cell line is present in the mesenchyme only in mosaic form. These findings suggest that in these atypical mosaicisms, there is a possible preferential pattern of distribution of the abnormal cell line.

**Table 5:** Description of atypical mosaic cases with two different mosaic abnormalities in chorionic villi and amniocytes.

Type of mosaicism	Trophoblast	Mesenchyme	Amniocytes
**CPM II**	46,XX[15]	47,XX,+9[12]/47,XX,+9,der(22)[2]	46,XX
	46,XX[11]	47,XX,+12,t(12;12)[4]/46,XX[26]	46,XX
	46,XX[10]	46,XX,fra(10)(q12) *	46,XX
**CPM III**	46,XX,add(8)[14]	46,XX,add(2)[13]	46,XX
	46,X,+mar[20]	45,X[9]	46,XY
**TFM V**	46,XY[14]	47,XY,+7[3]/46,XY[9]	mos 45,X[9]/46,XY[41]
**TFM VI**	46,X,der(Y)[15]	46,X,+mar[3]/45,X[2]/47,XX,+mar[3]/46,XX[24]/46,XY[3]	mos 45,X[40]/46,XY[11]
	46,XX,add(13)[16]	46,XX,add(13)[3]/46,XX[7]	46,XX,inv(13)(q11.1q32.1)dn.ish inv(13)(q11.1q32.1)dn(D13Z1/D21Z1+,D13S319+,LAMP1+,D13S1160+).arr(1-22,X)x2
	46,XY,r(22)[19]	45,XY,der(21;22)[5]	46,XY,r(22)(p11;q13)
	46,XY,add(7)[15]	46,XY,del(7)[15]	46,XY,del(7)(q32).ish del(7)(q32)(ELN+,LIMK1+,D7S613+,D7S486+,D7S522+,D7S427−)
	46,XX,add(6)[12]	47,XX,6ps,+mar[11]	46,XX,del(6)(p25.3)

* 5/19 Metaphases with fra(10); CPM = Confined Placental Mosaicism; TFM = True Fetal Mosaicism.

## 6. Risk of Fetal Uniparental Disomy (UPD) after the Detection of a Mosaic Abnormality Involving an Imprinted Chromosome

The frequency of UPD involving an imprinted chromosome (2, 6, 7, 11, 14, 15, 16, and 20) associated with CPM was calculated to be 0.01% in the overall diagnostic experience with CVS (5/52673). Six cases with UPD were found out of 243 investigated cases (2.5%). All of these cases derived from a mosaic cell line in CVS with a trisomy or a supernumerary marker chromosome or a structural abnormality predisposing to the occurrence of UPD (Table 6). 

**Table 6:** Description and incidence of uniparental disomies (UPD).

Type of abnormality	No. investigated cases	No. UPD	Type of CPM or TFM	UPD incidence (%)
trisomy 2	62	-	-	-
trisomy 7	60	-	-	-
trisomy 6	2	-	-	-
trisomy 11	3	-	-	-
trisomy 14	10	2	2 (CPM type I)	20
trisomy 15	24	1	1 (CPM type II)	4.2
trisomy 16	17	3	2 (CPM type III) + 1 (TFM type VI)	17.6
trisomy 20	25	-	-	-
sSMC, others	40	-	-	-
**Total**	**243**	**6**	**5 CPM and 1 TFM**	2.5

CPM = confined placental mosaicism; TFM = true fetal mosaicism; sSMC = small supernumerary marker chromosome; Others = translocations, deletions, inversions involving imprinted chromosomes.

Notably, the two UPD14 were both CPM type I with a low level of trisomy 14 only in the cytotrophoblast. In both cases, the indication for prenatal diagnosis was advanced maternal age. 

Consequently, these two cases would have been undetected if only LTC (with or without QF-PCR, for common aneuploidies) was performed, as most worldwide laboratories now offer [76]. 

True fetal trisomy 2 mosaicism has been reported in 1 in 58,000 cases of second-trimester amniocentesis [77]. The reported prevalence of mosaic trisomy 2 in CVS provided by the combined data from 5 studies on a total of 259,022 cases of CVS is approximately 1 in 1420 (Table 7) ([62,73,77,78], present study). Trisomy 2 in CVS was never confirmed in the fetus. UPD investigation was performed in 116 cases of CPM for trisomy 2, and all showed a normal biparental inheritance (Table 7). 

Trisomy 7 is extremely rare at birth and is generally considered lethal in embryogenesis [79]. In amniocytes, trisomy 7 is frequently a cell culture artifact that is possibly derived from an undetected low level of trisomy 7 mosaicism in uncultured amniocytes and is interpreted as likely pseudomosaicism with normal fetal outcome [49,80]. The reported prevalence of mosaic trisomy 7 in CV provided by the combined data from 4 studies on a total of 214,048 cases of CVS is approximately 1 in 1260 (Table 8) ([73,81,82], present study). Trisomy 7 in CVS shows a very low rate of fetal confirmation, and UPD investigation in 76 cases of CPM for trisomy 7 provided evidence of 1 case of UPD7mat (Table 8). The prevalence of UPD7 associated with CPM for trisomy 7 was predicted to be approximately 1/30 [73]. However, in our series of combined data, it seems to be lower (1/76, 1.3%).

**Table 7:** Prevalence of mosaic trisomy 2 in chorionic villi and UPD2 in the fetus.

Study	Total No. of CVS sample	No. of CVS samples with trisomy 2	Prevalence of trisomy 2 in CV		No. of TFM with trisomy 2 after a mosaic in CV	No. of cases with UPD investigation	No. of UPD2 retrieved	Incidence of UPD in mosaic trisomy 2 in CV (%)
			%	1/*x*				
Wolstenholme, 1996	66,129	41	0.06	1613	na	na	na	na
Hahnemann and Vejerslev, 1997	92,246	11	0.01	8386	0	na	na	na
Sago *et al*., 1997	10,500	11	0.10	955	na	11	0	0
Sifakis *et al*., 2010	37,474	45	0.12	833	0	43	0	0
Present Study *	52,673	74	0.14	712	0	62	0	0
**Total**	**259,022**	**182**	**0.07**	**1423**	**0**	**116**	**0**	**0**

* Including cases published in Grati *et al.*, 2006; na = not available.

**Table 8:** Prevalence of mosaic trisomy 7 in chorionic villi and UPD7 in the fetus.

Study	Total No. of CVS sample	No. of CVS samples with trisomy 7	Prevalence of trisomy 7 in CV		No. of TFM with trisomy 7 after a mosaic in CV	No. of cases with UPD investigation	No. of UPD7 retrieved	Incidence of UPD in mosaic trisomy 7 in CV (%)
			%	1/*x*				
Wolstenholme, 1996	66,129	60	0.09	1102	na	na	na	na
Hahnemann and Vejerslev, 1997	92,246	32	0.03	2883	0	na	na	na
Sachs *et al*., 1990	3000	5	0.17	600	0	na	na	na
Kalousek *et al*., 1996	na	na	na	na	na	14	1	7.1
Present Study *	52,673	73	0.14	722	0	62	0	0
**Total**	**214,048**	**170**	**0.08**	**1259**	**0**	**76**	**1**	**1.3**

* Including cases published in Grati *et al.*, 2006; na = not available.

UPD investigation in sSMC mosaic and other structural balanced and unbalanced mosaic rearrangements predisposing to its occurrence shows in all cases a normal biparental inheritance. The reported incidence of UPD in undefined series of >3300 sSMC is 1.3% [33]. 

## 7. Molecular Techniques for Detection of Chromosomal Mosaicism: Fluorescence *in Situ* Hybridization Analysis (FISH), Quantitative Fluorescent Polymerase Chain Reaction (QF-PCR), and Chromosomal Microarrays (Array Comparative Genomic Hybridization, aCGH; Single Nucleotide Polymorphism Array, SNP Array)

FISH analysis using a specific subset of probes for chromosomes involved in common aneuploidies (13, 18, 21, X and Y) can be used to assess the presence of these conditions in homogeneous or mosaic form in prenatal samples [83,84,85,86,87]. The advantages of this method are that they can bypass the need for culture (projecting a realistic description of the constitution of the prenatal sample without culture bias), provide a rapid result, and enable the detection of the low level mosaicism that requires analysis of a large number of metaphase spreads. The primary limits are technical problems related to poor hybridization efficiency and maternal blood contamination, and that only a subset of specific aneuploidies are tested, thus investigating only the segment of chromosome to which the probe binds [85]. 

QF-PCR is a DNA-based test for the detection of common aneuploidies by the amplification of repeat sequences at specific polymorphic loci. These repeat sequences are amplified by PCR, and the labeled products are separated by fluorescent capillary electrophoresis. An allele pattern of two equal peaks within the same chromosomal region is diagnostic of two copies of the target region, whereas three peaks within the same chromosomal region or two peaks with a ratio of 2:1 are indicative of trisomy for the target region [88]. QF-PCR can detect homogeneous and mosaic trisomies 13, 18, and 21 >20%, sex chromosome aneuploidies and triploidies. These chromosome abnormalities account for 70%–90% of the clinically significant cytogenetic abnormalities in the population of pregnancies with no a-priori risk other than the general population risk determined by maternal age [88,89,90]. Consistent maternal cell contamination (>70%) can be detected by comparison of fetal and maternal alleles. 

Microarray analysis is another diagnostic tool that can be used to detect fetal chromosome imbalances. It allows a simultaneous and comprehensive identification of both microscopic and submicroscopic unbalanced abnormalities. It is essentially a simultaneous FISH experiment with thousands or millions of probes that interrogates the entire genome in one experiment. Gains or losses are easily and objectively identified together with their genomic location, and data can be re-analyzed later in pregnancy at a higher resolution [91]. Microarrays cannot identify balanced rearrangements, and polyploidies are not detected by many modalities of aCGH. 

The prospective NICHD clinical trial [92] identified (likely) pathogenic submicroscopic imbalances in 6% of fetuses with ultrasound abnormalities and normal karyotypes and in 1.7% of anatomically normal fetuses and normal karyotypes; copy number variants with uncertain clinical consequences were detected in 3.4% of normal-karyotype fetuses (1.8% classified as likely benign and 1.6% with a potential role for clinical significance). Incidental/unsolicited findings related to adult on-set conditions or known pathological conditions unrelated to the reason for the test can be detected in 1–2 per 1000 analyses by genome-wide array [93,94]. 

Trisomy 21 mosaicism ≥20% can be identified by aCGH, as demonstrated by creating artificially derived mosaic samples with 10%–50% trisomy 21 in increments of 10% [95]. SNP arrays allow for the detection of as low as 5% mosaicism involving i(12p) (tetrasomy 12p) associated with Pallister-Killian syndrome [96]. When microarray analysis is applied to fresh CVS, feto-placental mosaicism present in approximately 1%–2% of samples may pose analytical/interpretative challenges because the differentiation in cytotrophoblastic and mesenchymal tissue separately is lost when DNA is extracted. Rare mosaic dosage gains/losses may result in erroneous diagnostic conclusions due to the possible detection of clinically significant cryptic copy number variations (CNVs) confined to the cytotrophoblast [97,98].

## 8. Potential False Positive and False Negative Results Using only (Semi-)Direct Preparation (STC) or Long-Term Culture (LTC)

The gold standard for cytogenetic analysis of CVS is the combined approach of both direct preparation/short-term culture and long-term culture [7,23]. This combined approach is associated with a rate of false negative (FN) and positive (FP) results of <1/40,000 [69] and 1.56% [CPM type 1 + 2 + 3/true negative (normal results) + CPM type 1 + 2 + 3 = 773/48,739 + 773] (present study), respectively. When only STC or LTC results can be obtained due to low sample amount or culture failure, these risks increase. Several studies explored these risks but, the issue has not been definitely resolved [2,81,99] due to the dearth of or the heterogeneity of samples collected in multicentric studies. 

Our retrospective analysis is based on a large cytogenetic dataset that incorporates both the cytotrophoblast and mesenchymal core. For this reason our analysis allows us to estimate the frequency of potential FP and FN results with only one method. In particular, when performing only STC, (i) TFM type V is not identified, generating FN results with an empiric frequency of ~1:1000, in accordance with a previous estimation [2]; and (ii) non-mosaic CPM type I and III could generate FP results with an estimated empiric frequency of ~2.6%. When performing LTC alone, TFM type IV is not identified, generating FN results with an estimated empiric frequency of ~1:2200, including also possible misdiagnosis due to complete maternal cell contamination (MCC) in LTC, and undetectable UPD cases (mainly of chromosome 14, see Table 6) consequent to the presence of a mosaic trisomy in the cytotrophoblast. To reduce FN results due to consistent (>70%) maternal cell contamination, an MCC exclusion test by QF-PCR in female LTC comparing maternal and fetal alleles is advisable [76]. With only LTC, CPM type II and III with an NMA karyotype on LTC could generate FP results with an empiric estimated frequency of ~1.2%. 

## 9. Potential False Positive and False Negative Results with Non-invasive Prenatal Screening (NIPS) for Screening (NIPS) for Common Aneuploidies Due to Feto-Placental Mosaicism

The presence of cell-free DNA (cfDNA) in maternal circulation was described for the first time two decades ago [100], and recently, non-invasive prenatal screening (NIPS) for fetal aneuploidy using cell-free fetal DNA (cffDNA) has been available commercially [101,102]. The NIPS test is erroneously popularized as “fetal” screening because over 99% of the cffDNA circulating in maternal plasma originates from apoptosis of the cells of the outer layers of the placenta: cytotrophoblasts and syncytiotrophoblasts [103,104,105]. Hence, we recently proposed the term “cffDNA” for sequences derived from amniotic fluid and the term cell free placental DNA, “cfpDNA”, when referring to the actual NIPS [68]. 

NIPS can report discordant findings compared to the fetal karyotype [106]. These may result from different phenomena such as a vanishing twin or co-twin demise [107], non-mosaic maternal chromosome abnormality [108], maternal metastatic disease [109], low fetal fraction [110], and feto-placental mosaicism [111,112]. Regarding this last phenomenon, mosaics in which the cytotrophoblast is cytogenetically discrepant from the fetus are sources of FP and PN results: CPM type I and III with an abnormal cytotrophoblast and normal amniocytes can be potential sources of FP, while TFM type V with a normal cytotrophoblast and abnormal amniocytes can be a potential source of FN results. The prediction of the contribution of feto-placental mosaicisms to FP and FN rates for each chromosome abnormality that was identified or can be identified by NIPS (trisomies 13, 18, 21, monosomy X, 47,+i(13q), 47,+i(21q), 47,XXX/XXY/XYY, mos 45,X/47,XXX and partial imbalances of the targeted chromosomes), is based on the retrospective analysis of our cytogenetic dataset of 52,673 CVS containing results for the cytotrophoblast and mesenchyme for each case [68]. This analysis found a FP rate of approximately 1/1100 of normal cases and a FN rate approximately 1/61 in abnormal karyotypes. This evaluation indicates that FP and FN results may be explained in part by the underlying physiologic placental-fetal genetic mechanisms. This could be minimum estimates for the FP and FN rates because it assumes that karyotyping identifies all such cases. In fact, there is data that suggests the placenta can be quite variable from site to site [113].

## 10. Conclusions

The most significant change in prenatal practice in recent years has been the advent of microarrays and NIPS [94,114,115]. The use of microarrays is currently being debated in regards to its possible replacement of conventional karyotyping in all-risk pregnancies [116,117] (see also paragraph 7). Using microarrays on native CVS, the morphology of placental cells (trophoblast and mesenchyme) and the distribution of the abnormal cell line cannot be assessed. For these reasons, the risk of fetal involvement cannot be reliably estimated. In particular, there are three critical situations: (i) FN cases can happen when a mosaic in CVS is not detected due to low percentages of abnormal cells in the cytotrophoblast and/or mesenchyme; (ii) FP results might be due to the inability of the molecular approach to discriminate between a generalized high level MA and a homogeneous abnormality; and (iii) the possibility of a FP result regarding a clinically significant cryptic copy number variation (CNV) confined to the cytotrophoblast, as already demonstrated by Karampetsou *et al*. [98]. When assessing data from the paper published by Karampetsou *et al*. [98], and from the present review, it is important to note that the introduction of the detection of some recurrent microdeletion disorders syndrome in cfpDNA might give FP and FN results because the target of NIPS is the trophoblast. In addition, when a chromosomal microarray of native CVS or NIPS on maternal plasma gives a positive result for a pathogenic CNV, a follow-up FISH analysis of metaphases either from the cytotrophoblast and from the mesenchyme is required. As an alternative, an AF confirmatory analysis, especially in absence of suggestive echographic findings, should be offered because of the underlying physiologic placental-fetal genetic mechanisms. Prospective studies are necessary to assess the incidence of confined placental and true fetal mosaicism for pathogenic structural cryptic imbalances to define the associated FP and FN rates. 

While prenatal diagnosis is destined to incorporate microarrays and NIPS (and, perhaps, in the future, also NGS) as routine practice, the substantial cytogenetic diagnostic experience with CVS presented in this review can be helpful and still provide reference data that will be applicable. The results on CVS ([16,17,18,19,20,21,22,23], present study) still have a role in evaluating the limits and advantages of the new technologies, and the findings can be integrated into pre- and post-test counseling. Only a deep understanding of the biology and genetic physiology of the placenta can ensure positive and appropriate integration of new technologies into clinical prenatal care processes and can support patient choice. 

## Supplementary Files

Supplementary File 1

## Abbreviations

aCGH: array comparative genomic hybridization
AF: amniotic fluid
cffDNA: cell free fetal DNA
cfpDNA: cell free placental DNA
CNV: copy number variation
CPM: confined placental mosaicism
CVS: chorionic villous sample
FISH: fluorescence *in situ* hybridization
FN: false negative
FP: false positive
h/UPDmat/pat: maternal/paternal heterodisomy/isodisomy in uniparental disomy condition
IUGR: intrauterine growth restriction
LTC: long term culture
MA: mosaic abnormality
MCC: maternal cell contamination
NDJ: non disjunction
NIPS: non-invasive prenatal screening
NGS: next generation sequencing
NMA: non mosaic abnormality
PNGR: postnatal growth retardation
QF-PCR: quantitative fluorescence polymerase chain reaction
STC: short term culture
SNP: single nucleotide polymorphism
STR: short tandem repeat
TFM: true fetal mosaicism
UPD: uniparental disomy
