# Supplementary figures and images for: Chromosomal Mosaicism in Human Feto-Placental Development: Implications for Prenatal Diagnosis
Source: J Clin Med. 2014 Jul 24;3(3):809–37. doi: 10.3390/jcm3030809 (PMC4449651; doi:10.3390/jcm3030809)

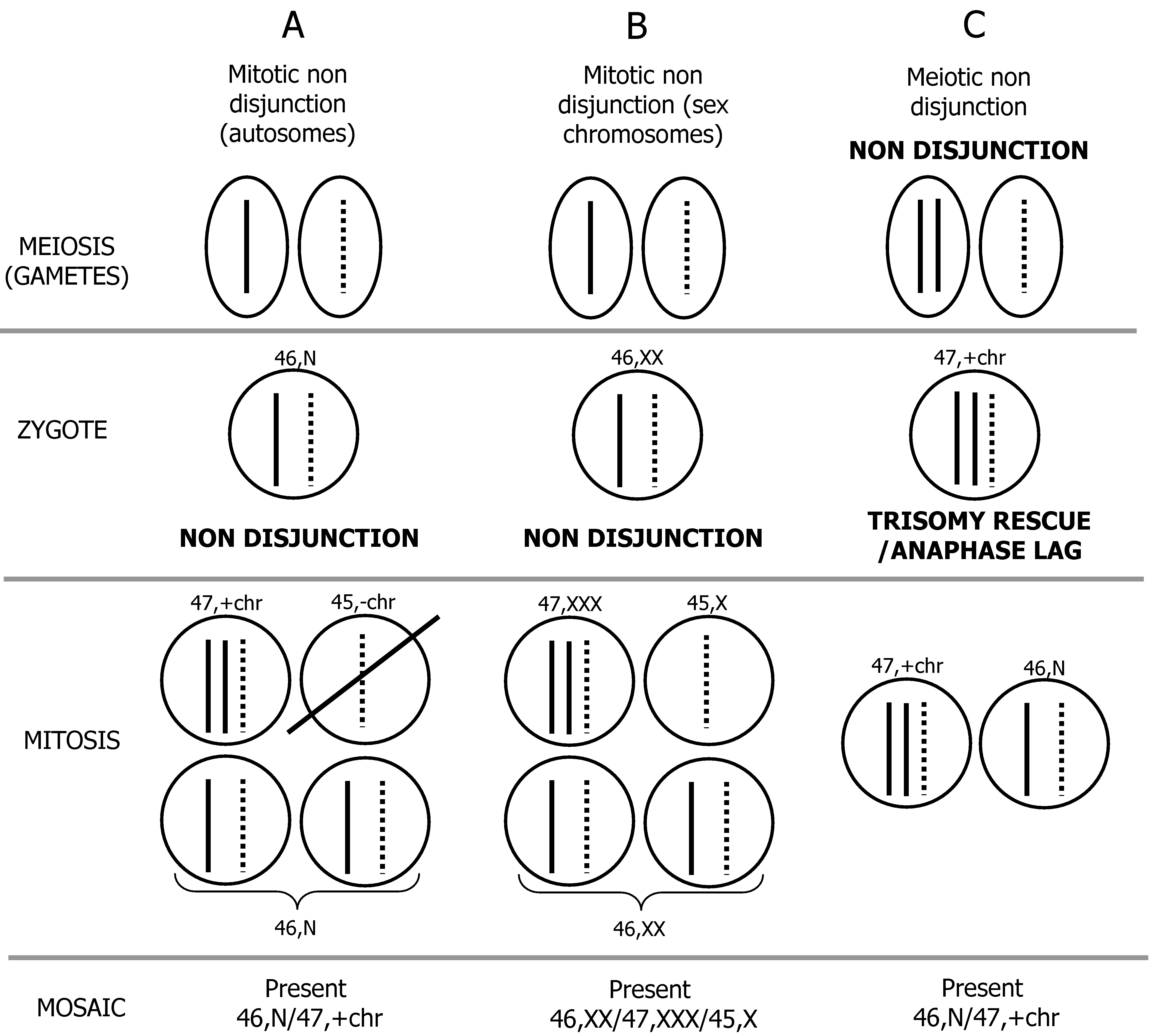

Supplement: Supplementary File 1 [file jcm-03-00809-s001.jpg]
